# Erector Spinae Plane Block With Selective Use of Adjunct Fascial Plane Blocks for Analgesia in Latissimus Dorsi Flap Breast Reconstruction: A Case Series With Mid-term Follow-Up

**DOI:** 10.7759/cureus.104300

**Published:** 2026-02-26

**Authors:** Chiara Angeletti, Laura Valente, Luca Gentili, Massimiliano Luca D'Agostino, Stefano Troili, Valentina Arcangeli, Federica Venturoni, Giustino Varrassi, Guido Torresini

**Affiliations:** 1 Operative Unit of Anaesthesiology, Intensive Care, and Pain Medicine, Civil Hospital Giuseppe Mazzini, Teramo, ITA; 2 Department of Anesthesia and Intensive Care Unit, Santa Maria Goretti Hospital, Latina, ITA; 3 Department of Clinical Medicine, Public Health, and Life Sciences (MESVA), University of L'Aquila, L'Aquila, ITA; 4 Department of Pain Medicine, Fondazione Paolo Procacci, Rome, ITA; 5 Department of Plastic Surgery, Civil Hospital Giuseppe Mazzini, Teramo, ITA

**Keywords:** breast reconstruction, erector spinae plane block, latissimus dorsi flap, postoperative analgesia, regional anesthesia

## Abstract

Background

Autologous breast reconstruction using the latissimus dorsi (LD) flap remains a reliable option in selected patients but is associated with significant postoperative pain involving both the anterior chest wall and the posterior donor site. Optimizing perioperative analgesia while minimizing opioid consumption is a key component of enhanced recovery pathways. Ultrasound-guided fascial plane blocks, particularly the erector spinae plane block (ESPB), have emerged as promising alternatives to traditional neuraxial techniques.

Objective

This study aims to describe the clinical experience and preliminary outcomes associated with ultrasound-guided ESPB, with or without an adjunct parasternal intercostal plane (PIP) block, in patients undergoing LD flap breast reconstruction as part of a multimodal analgesic strategy.

Methods

This single-center retrospective observational case series included six adult female patients undergoing elective autologous breast reconstruction with an LD flap between March 2022 and December 2024. All patients received ultrasound-guided ESPB; in selected cases, a PIP block was added based on anatomical considerations. Postoperative pain was assessed using the Numeric Rating Scale (NRS) at rest and during movement up to 48 hours. Opioid consumption, postoperative nausea and vomiting, mobilization, and block-related complications were recorded. Mid-term follow-up at 6 and 12 months was conducted via structured telephone interviews that assessed persistent pain, functional recovery, complications, and patient satisfaction. Results are reported using descriptive statistics.

Results

All patients achieved low postoperative pain scores, with NRS values of 0 at rest immediately after surgery. Dynamic pain scores remained low during the first 48 hours (median NRS ≤3), and rescue opioid analgesia was required in one patient. No postoperative nausea, vomiting, or block-related complications were observed, and all patients mobilized within 6-12 hours. At 6- and 12-month follow-up, no patient reported persistent pain requiring ongoing analgesic therapy, shoulder mobility was preserved, and overall patient satisfaction was high.

Conclusions

In this case series, ESPB within a multimodal analgesic approach was associated with favorable acute and mid-term pain outcomes, minimal opioid requirements, preserved shoulder function, and high patient satisfaction following LD flap breast reconstruction. The selective use of adjunct anterior chest wall blocks reflects an individualized, anatomy-driven strategy. These findings support the feasibility of ESPB-centered analgesic pathways in this surgical setting and warrant confirmation in larger prospective studies. These findings should be interpreted as hypothesis-generating rather than confirmatory.

## Introduction

Breast reconstruction is a crucial component of the multidisciplinary management of breast cancer, contributing not only to physical restoration but also to psychological well-being and overall quality of life.

Globally, breast reconstruction rates continue to increase, with more than 40% of women undergoing mastectomy in Europe and North America receiving immediate or delayed reconstruction. In the United States, more than 160,000 breast reconstruction procedures are performed annually. In Europe, autologous techniques remain widely used, particularly in complex or salvage settings [1-4].

Beyond post-mastectomy reconstruction, the latissimus dorsi (LD) flap is also widely used for reconstruction of regional defects involving the chest wall, axilla, and upper limb, supporting its role as a versatile workhorse flap across anatomical districts.

Among autologous reconstruction techniques, the LD flap continues to play a relevant role, particularly in patients who are not suitable candidates for abdominally based flaps, in salvage procedures, or when additional soft-tissue coverage is required following radiotherapy or implant failure [5,6]. Owing to its reliable vascular supply through the thoracodorsal system and its versatility, the LD flap remains a dependable reconstructive option in complex clinical scenarios.

Despite advances in surgical techniques, LD flap breast reconstruction is still associated with significant postoperative pain. This pain results from extensive thoracic wall dissection, mobilization of a large muscle mass, and the involvement of both anterior and posterior thoracic regions. Inadequately controlled pain may delay early mobilization, prolong hospital stay, increase opioid consumption, and contribute to the development of chronic post-surgical pain, ultimately impairing functional recovery and patient satisfaction [7].

Traditionally, postoperative analgesia in breast reconstruction has relied on systemic opioids and neuraxial regional techniques, such as thoracic epidural analgesia (TEA) and thoracic paravertebral block (PVB). Although effective, these approaches are limited by technical complexity, contraindications, and the risk of complications, including hypotension, urinary retention, and pneumothorax [8,9]. These limitations have encouraged the development of alternative strategies within multimodal and opioid-sparing analgesic protocols.

In recent years, ultrasound-guided fascial plane blocks have gained increasing attention as safer and more versatile alternatives for perioperative pain management. Among these techniques, the erector spinae plane block (ESPB) has emerged as a promising regional anesthetic approach since its initial description by Forero et al. in 2016 [10]. ESPB involves the injection of local anesthetic into the fascial plane deep to the erector spinae muscle, allowing cranio-caudal spread and potential diffusion toward the paravertebral space. Anatomical and imaging studies have demonstrated that ESPB can provide multi-segmental analgesia by affecting both dorsal and ventral rami, making it particularly suitable for thoracic and breast surgery [11].

Clinical studies have shown that ESPB effectively reduces postoperative pain scores and opioid consumption in breast surgery, with a favorable safety profile when compared with traditional regional techniques [12-15]. Its technical simplicity and low complication rate have led to its increasing incorporation into Enhanced Recovery After Surgery (ERAS) pathways for breast procedures [16]. However, most available evidence focuses on mastectomy and implant-based reconstruction.

LD flap reconstruction represents a distinct surgical context, characterized by extensive posterior thoracic dissection and donor-site morbidity, which may not be fully addressed by anterior chest wall blocks alone. For this reason, combined or alternative regional techniques, such as serratus anterior plane block or combined interfascial approaches, have been proposed to achieve more comprehensive analgesic coverage [17,18].

To date, literature specifically addressing the use of fascial plane blocks in LD flap reconstruction remains limited and heterogeneous, consisting mainly of case reports and small case series. Siow et al. reported effective postoperative analgesia using ESPB in LD flap reconstruction, highlighting reduced opioid requirements and satisfactory pain control [19]. Similar observations have been reported in small observational studies combining ESPB with other interfascial techniques, although standardized protocols and high-quality comparative data remain lacking [20,21].

Given the increasing use of autologous breast reconstruction and the growing emphasis on opioid-sparing perioperative strategies, further clinical evidence is needed to clarify the role of regional anesthesia in this specific surgical population. Real-world clinical experience may help define optimal analgesic approaches and improve postoperative outcomes in patients undergoing LD flap breast reconstruction.

The aim of this single-center observational case series is to evaluate the effectiveness of ultrasound-guided fascial plane blocks as part of a multimodal analgesic approach in patients undergoing LD flap breast reconstruction, focusing on postoperative pain control, opioid consumption, functional recovery, and mid-term outcomes.

## Materials and methods

Study design and patient selection

This study is a single-center, retrospective observational case series conducted at Giuseppe Mazzini Hospital (Teramo, Italy). Six adult female patients undergoing elective autologous breast reconstruction with an LD flap between March 2022 and December 2024 were included. All consecutive patients undergoing LD flap reconstruction during the study period who met the inclusion criteria were included. Cases were identified through institutional surgical logs, and perioperative data were extracted from electronic medical records to minimize recall bias.

The limited sample size reflects the low volume of LD flap reconstructions performed at our institution, where this technique is reserved for selected cases and is not routinely performed. All included cases met predefined inclusion criteria and had complete perioperative clinical data and available mid-term follow-up at the time of manuscript preparation.

This study was conducted as a retrospective observational case series based on routine clinical practice. According to institutional policy, formal ethics committee approval was not required for retrospective case series without experimental interventions. Written informed consent for the anesthetic procedures, data collection, and publication was obtained from all patients.

This case series was prepared in accordance with the CARE reporting guidelines. The inclusion and exclusion criteria are presented in Table 1.

**Table 1:** Inclusion and exclusion criteria ESPB, erector spinae plane block

Inclusion criteria	Exclusion criteria
Age ≥18 years	Refusal or inability to provide informed consent
Elective autologous breast reconstruction with latissimus dorsi flap	Contraindications to regional anesthesia
Use of ultrasound-guided ESPB with or without adjunct chest wall blocks	Known allergy to local anesthetics or adjuvant drugs
Availability of complete perioperative clinical data	Infection at the needle insertion site
Ability to complete postoperative and mid-term follow-up	Coagulopathy or therapeutic anticoagulation not compatible with regional blocks
-	Chronic opioid use or pre-existing chronic pain unrelated to surgery
-	Incomplete clinical data or loss to follow-up

Regional anesthesia techniques

All regional blocks were performed preoperatively, before induction of general anesthesia, with patients awake or lightly sedated. All regional anesthesia procedures were performed under ultrasound guidance using a 13-6 MHz linear transducer (Sonosite Europe, Amsterdam, The Netherlands) and a 90-mm, 22G echogenic needle. Skin antisepsis was performed using 2% chlorhexidine.

The local anesthetic solution consisted of levobupivacaine 0.25% or ropivacaine 0.25%, selected according to patient characteristics and drug availability. Maximum recommended doses were respected (levobupivacaine ≤2 mg/kg; ropivacaine ≤3 mg/kg). Adjuvants included clonidine or dexmedetomidine (1 μg/kg); intravenous dexamethasone (4-8 mg) was administered in selected cases as part of multimodal analgesia.

For the ESPB, the transducer was positioned parasagittally approximately 3 cm lateral to the T6 or T8 spinous process, depending on surgical side and anatomy. The needle was advanced in-plane to the fascial plane deep to the erector spinae muscle. After hydrodissection with saline, 30-40 mL of a local anesthetic solution was injected per side. ESPB was performed with the patient in the sitting position.

In selected cases, an additional parasternal intercostal plane (PIP) block was performed to enhance analgesia of the anterior chest wall. With the patient in the supine position, the transducer was placed parallel to the sternum, and a local anesthetic was injected into the fascial plane between the pectoralis major and the external intercostal muscle after hydrodissection. The decision to add a PIP block was based on anticipated anterior chest wall involvement, implant placement, and the anesthesiologist's clinical judgment and was not applied routinely to all patients.

**Figure 1:**
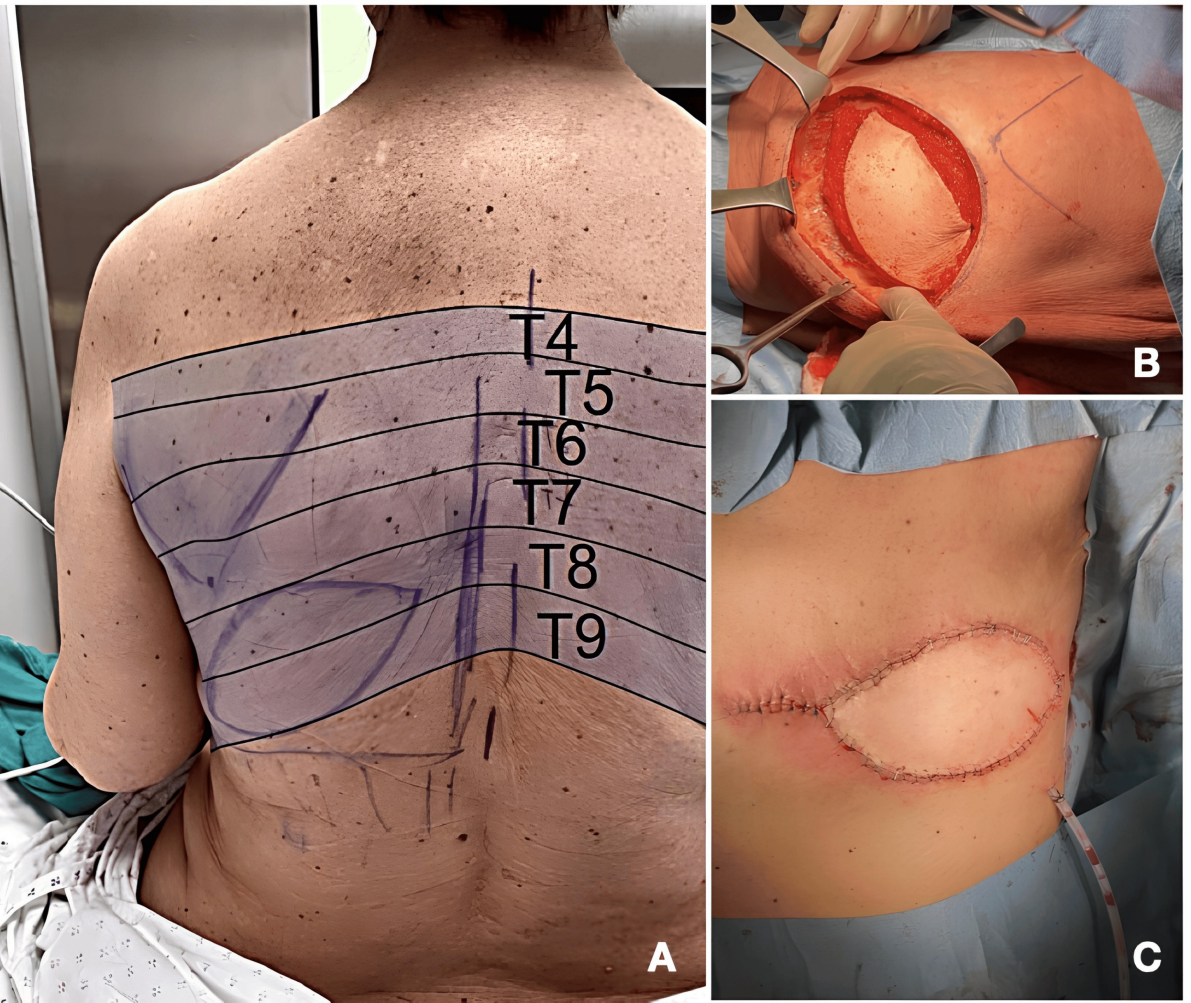
Dermatomal coverage and surgical stages of latissimus dorsi flap harvest in relation to regional anesthesia strategy (A) Posterior thoracic dermatomal distribution (T4–T8) corresponding to the expected sensory territory involved in latissimus dorsi flap harvest. This area represents the primary target for regional anesthetic coverage. (B) Intraoperative view of latissimus dorsi flap elevation demonstrating muscle plane dissection within the mapped dermatomal region. (C) Donor site after flap harvest showing elliptical skin closure and placement of surgical drain, highlighting the postoperative pain-generating area. All images were obtained intraoperatively with informed patient consent.

An immediate post-procedural ultrasound check was performed to assess local anesthetic spread, particularly for ESPB. Sensory block was evaluated using a pinprick test approximately 30 minutes after block placement (Figure 1A).

No local anesthetic infiltration at the surgical or donor site was performed by the surgeon in the cases included in this series.

Variations in local anesthetic type and adjuvant use reflected real-world clinical practice and were based on patient characteristics and drug availability.

Anesthesia and surgical management

General anesthesia was induced using propofol (2 mg/kg), fentanyl 100 μg intravenously at induction in all patients, and rocuronium (0.6 mg/kg), followed by endotracheal intubation (7.0-8.0 mm). The intraoperative opioid regimen reflected institutional practice and was not modified for study purposes.

Standard intraoperative monitoring included heart rate, invasive arterial blood pressure, peripheral oxygen saturation, electrocardiography, end-tidal CO₂, bispectral index (BIS), and train-of-four (TOF).

Anesthesia was maintained with sevoflurane (minimum alveolar concentration 0.4-0.5), titrated to achieve a BIS, targeted between 40 and 50, and a continuous remifentanil infusion (0.1-0.15 μg/kg/min), titrated according to hemodynamic parameters and BIS monitoring.

All patients underwent immediate LD flap breast reconstruction (Figures 1B, 1C). In selected cases, tissue expanders were placed according to surgical indication. Surgical technique followed standard institutional practice. Surgical indications for implant removal and selection of reconstructive techniques were established by the plastic surgery team and were not analyzed as study variables, as the focus of this case series was anesthesiological management and pain outcomes.

Postoperative analgesia

Postoperative analgesia followed routine clinical practice. Paracetamol (1 g) and ketorolac (30 mg) were administered intravenously approximately 30 minutes before the end of surgery. Postoperative analgesics were prescribed on an as-needed basis according to patient-reported pain intensity and the clinical judgment of the attending ward physician. No fixed postoperative analgesic protocol was applied.

Rescue analgesia included intravenous paracetamol (1 g) for Numeric Rating Scale (NRS) scores >3. In cases of uncontrolled pain (NRS >6-8), intravenous tramadol (100 mg) or a morphine elastomeric infusion was administered at the discretion of the treating physician.

Pain assessment and in-hospital outcomes

Acute postoperative pain was assessed using the NRS (0-10) at rest and during mobilization. Dynamic pain assessment included pain during mobilization and, when applicable, during deep breathing. In the operating room and recovery area, NRS scores were recorded by anesthesia nurses or anesthesia residents as part of routine postoperative monitoring. During hospitalization, NRS scores were recorded by anesthesia nurses in the recovery room and by ward nurses during postoperative care, in accordance with standard institutional protocols, and were subsequently extracted retrospectively from clinical charts. Pain assessment was part of standardized institutional postoperative monitoring and was not modified for study purposes.

NRS assessments were collected at predefined time points (0, 3, 6, 12, 24, and 48 hours postoperatively). Data for this case series were retrospectively extracted from clinical records. Opioid consumption, postoperative nausea and vomiting (PONV), early mobilization, and block-related complications were also recorded.

Mid-term follow-up

Midterm follow-up was conducted at 6 and 12 months postoperatively via structured telephone interviews conducted as part of routine clinical practice. Interviews were conducted by a member of the clinical team using a standardized set of questions.

Patients were asked about (1) persistent postoperative pain and, when present, pain intensity quantified using the NRS and need for ongoing analgesic therapy; (2) shoulder function and limitations in daily activities, categorized as full range of motion, mild stiffness, or functional limitation, and need for physiotherapy beyond the early postoperative period; (3) postoperative complications, including infectious events; and (4) satisfaction with postoperative pain management and overall recovery, assessed using a Likert-type scale (very satisfied, satisfied, neutral, dissatisfied).

Follow-up assessments were based on non-validated patient-reported measures, which may be subject to recall bias and social desirability bias.

Ethical considerations

This study is a retrospective observational case series based on routine clinical practice. Written informed consent for the procedures, data use, and publication was obtained from all patients. According to institutional policy, formal ethics committee approval was not required for retrospective case series using anonymized data and involving no deviation from the standard of care. The study was conducted in accordance with the Declaration of Helsinki.

Statistical analysis

Given the descriptive nature of this case series, results are reported using descriptive statistics, including absolute numbers, percentages, medians, and ranges. No inferential statistical analyses were performed.

## Results

Given the descriptive nature of this observational case series and the small sample size (n = 6), results are presented using descriptive statistics, including absolute numbers, percentages, medians, and ranges. No inferential statistical analyses were conducted.

Patient and surgical characteristics

Six female patients underwent autologous breast reconstruction using the LD flap between March 2022 and December 2024. The median age was 61.5 years (range 45-66), and the median body mass index (BMI) was 24.9 kg/m² (range 21.6-28.3). Four patients (66.7%) were classified as ASA (American Society of Anesthesiologists) II and two (33.3%) as ASA III. Previous breast surgery was reported in four patients (66.7%), most commonly mastectomy.

Reconstruction was unilateral in five patients (83.3%; four right-sided and one left-sided) and bilateral in one patient (16.7%). Immediate LD flap reconstruction was performed in all cases, with tissue expanders placed in four patients (66.7%). The median operative time was 280 minutes (range 120-340). The median length of hospital stay was five days (range 5-6) (Table 2).

**Table 2:** Patient and surgical characteristics BMI, body mass index; ASA, American Society of Anesthesiologists physical status classification; LD, latissimus dorsi; LN, lymph node; LOS, length of hospital stay; Op time, operative time

Case	Age (years)	BMI (kg/m^2^)	ASA	Laterality	Prior breast surgery	Reconstruction/procedure	Implant/expander	Op time (min)	Drains	LOS (days)
1	59	25.3	2	Left	Left mastectomy	Implant removal + LD flap	None	220	1	5
2	45	22.0	2	Bilateral	Left quadrantectomy	Bilateral mastectomy + left LD flap reconstruction	Bilateral expander	320	4	6
3	65	28.3	2	Right	Right quadrantectomy	Right mastectomy + LN biopsy + sternal liposuction + LD flap reconstruction + symmastia correction	None	340	2	6
4	66	24.5	3	Left	Left mastectomy	Left LD flap	Implant	120	1	5
5	54	21.6	2	Right	Right mastectomy	Right LD flap	Implant	330	2	5
6	64	25.4	3	Left	Left mastectomy	Implant removal + LD flap reconstruction	Implant	240	2	5

Regional anesthesia techniques

All patients (100%) received ultrasound-guided regional anesthesia. ESPB was performed unilaterally or bilaterally in all cases. In two patients (33.3%), an additional PIP block was administered, based on anatomical considerations, to enhance coverage of the anterior chest wall. Due to the limited number of cases, no comparative assessment of its additive effect was performed.

ESPB was performed at the T6 or T8 level, depending on the surgical side and patient anatomy. The local anesthetic solution consisted of levobupivacaine 0.25% or ropivacaine 0.25%, with a total injected volume of 30-40 mL per side. Adjuvants included clonidine or dexmedetomidine (1 μg/kg), and intravenous dexamethasone was administered in selected cases as part of multimodal analgesia.

ESPB was performed with the patient in the sitting position, while the PIP block was performed in the supine position. Sensory block was assessed using a pinprick test approximately 30 minutes after block placement. No block failures or immediate block-related complications were observed (0%) (Table 3).

**Table 3:** Regional anesthesia details ESPB, erector spinae plane block; PIP, parasternal intercostal plane block; LAP, local anesthetic placement; US, ultrasound; T, thoracic vertebral level

Case	Block(s)	Side LAP	Level	US spread check	Position	Incision after block (min)	Local anesthetic	Volume (mL)	Adjuvants	Catheter
1	ESPB	Left	T8	T4–T10	Sitting	50	Levobupivacaine 0.25% (75 mg)	30	Dexmedetomidine 60 mcg + Dexamethasone 4 mg	No
2	Bilateral ESPB	Left	T8	T5–T11	Sitting	40	Ropivacaine 0.25% (150 mg)	30	Clonidine 50 mcg + Dexamethasone 8 mg	No
3	ESPB + PIP block	Right	T7	T5–T10	Sitting/supine	55	Levobupivacaine 0.25% (100 mg)	30 + 10	Clonidine 70 mcg	No
4	ESPB	Left	T6	T6–T9	Sitting	58	Levobupivacaine 0.25% (75 mg)	30	Dexmedetomidine 70 mcg + Dexamethasone 4 mg	No
5	ESPB + PIP block	Right	T8	T6–T11	Sitting/lateral	35	Levobupivacaine 0.25% (100 mg)	30 + 10	Dexmedetomidine 70 mcg + Dexamethasone 4 mg	No
6	ESPB	Left	T7	T5–T9	Sitting	45	Levobupivacaine 0.25% (75 mg)	30	Clonidine 65 mcg + Dexamethasone 4 mg	No

Postoperative outcomes

Postoperative pain was assessed using the NRS at rest and during movement. At 0 hours, NRS scores were 0 in all patients (100%). At 12 hours postoperatively, dynamic NRS scores ranged from 0 to 5, with a median value of 2.5. Between 24 and 48 hours, dynamic pain scores ranged from 0 to 4 (median 3).

Rescue non-opioid analgesia was required in most patients, while opioid rescue was needed in only one case.

Rescue analgesia with intravenous paracetamol (1 g) was administered when NRS exceeded 3. Only one patient (16.7%) required intravenous tramadol (100 mg) at 48 hours. No patients (0%) experienced postoperative nausea or vomiting. All patients (100%) were able to mobilize within 6-12 hours after surgery. No block-related complications were recorded during the hospital stay (Table 4).

**Table 4:** Early postoperative outcomes NRS, Numeric Rating Scale; dyn, dynamic (during mobilization); PONV, postoperative nausea and vomiting

Case	Preventive analgesia	NRS T0 (rest/dyn)	NRS 12h (rest/dyn)	NRS 24h (rest/dyn)	NRS 48h (rest/dyn)	Rescue analgesia	PONV	Block-related complications	Early mobilization (6–12h)
1	Paracetamol 1 g + ketorolac 30 mg	0/0	0/3	0/3	0/3	Paracetamol 1 g	No	None	Yes
2	Paracetamol 1 g + ketorolac 30 mg	0/0	0/3	0/2	0/3	Paracetamol 1 g (day 2–3)	No	None	Yes
3	Paracetamol 1 g + ketorolac 30 mg	0/0	0/2	0/2	0/3	Not required	No	None	Yes
4	Paracetamol 1 g + ketorolac 30 mg	0/0	0/5	0/3	0/3	Ketorolac + tramadol (then paracetamol)	No	None	Yes
5	Paracetamol 1 g + ketorolac 30 mg	0/0	0/2	0/3	0/4	Paracetamol 1 g (day 2–4)	No	None	Yes
6	Paracetamol 1 g + ketorolac 30 mg	0/0	0/0	0/3	0/4	Paracetamol 1 g (day 2)	No	None	Yes

Mid-term follow-up outcomes

Several patients reported mild intermittent donor-site symptoms, such as tightness or discomfort, which did not meet criteria for chronic pain and did not require analgesic therapy.

All patients (100%) completed structured telephone follow-up at both 6 and 12 months postoperatively. No patient reported persistent postoperative pain at rest requiring ongoing analgesic therapy, with NRS scores of 0/10 at rest. Mild intermittent discomfort, characterized by pulling or tightness at the donor site or shoulder, was reported by some patients but did not require analgesic treatment.

All patients (100%) reported a full shoulder range of motion. Three patients (50%) required physiotherapy beyond the early postoperative period. One patient (16.7%) developed a donor-site scar infection caused by *Streptococcus*, which resolved with conservative treatment.

Overall, patient satisfaction was high: four patients (66.7%) reported being "very satisfied," and two patients (33.3%) reported being "satisfied" with postoperative pain management and recovery. At 12 months, no patient required long-term analgesic therapy or additional interventions related to pain or functional impairment (Table 5).

**Table 5:** Telephone follow-up (6–12 months) ROM, range of motion; PO, postoperative

Case	6-month status	12-month status	Shoulder mobility	Physiotherapy	Infection/complications	Satisfaction
1	Donor-site tension/paresthesia	No pain/allodynia/hyperalgesia	Full ROM, normal function	PO period	None	Very satisfied
2	No pain; no analgesics	No pain; mild stiffness/tightness without functional impairment	Full ROM, normal function	No	None	Very satisfied
3	Healing completed after infection	Occasional weather-related pain; no analgesic therapy	Full ROM, normal function	PO period	Scar infection (*Streptococcus*)	Very satisfied
4	No pain; no analgesics	No pain; no pain medication	Full ROM, normal function	PO period	None	Satisfied
5	Stiffness/tightness	Mild intermittent donor-site pulling/numbness	Full ROM, normal function	No	None	Satisfied
6	No pain; no analgesics	Occasional muscle cramping/weakness	Full ROM, normal function	PO period	None	Very satisfied

Three patients required physiotherapy beyond the early postoperative period, reflecting the known functional impact of LD flap harvest.

## Discussion

Autologous breast reconstruction represents a cornerstone of post-mastectomy restoration, offering durable aesthetic outcomes and substantial psychosocial benefits when compared with implant-based approaches. Among autologous techniques, the LD flap continues to play a relevant role, particularly in patients who are not suitable candidates for abdominally based free flaps, require salvage procedures after implant failure, or need additional soft-tissue coverage in irradiated or complex clinical scenarios. Its reliable vascular supply through the thoracodorsal system and broad surface area underpin its ongoing use in challenging reconstructive settings [6,22].

Despite its reconstructive advantages, LD flap surgery is consistently associated with significant postoperative pain. This is related to the presence of two distinct pain generators: the anterior thoracic recipient site and the extensive posterior donor-site dissection. Inadequate pain control in this context may delay mobilization, impair respiratory mechanics, increase opioid consumption, and negatively affect postoperative recovery and patient satisfaction [23]. These considerations align closely with the ERAS principles, which emphasize opioid-sparing analgesia, early mobilization, and optimization of patient-centered outcomes [24,25].

Traditionally, TEA and PVB have been regarded as effective regional anesthesia techniques for breast and thoracic surgery. However, their use may be limited by invasiveness, technical complexity, and potential complications such as hypotension, urinary retention, and pneumothorax, as well as relative contraindications in anticoagulated patients [8,9]. These limitations have driven increasing interest in ultrasound-guided fascial plane blocks as potentially safer and more flexible alternatives within multimodal analgesic strategies [26,27].

Among these techniques, the ESPB has gained considerable attention since its first description in 2016 [10]. ESPB is generally considered easy to perform under ultrasound guidance and is associated with a favorable safety profile. Anatomical and radiological studies suggest that injection of local anesthetic deep to the erector spinae muscle allows cranio-caudal spread with variable extension toward the dorsal and ventral rami and, in some cases, the paravertebral space, resulting in multisegmental thoracic analgesia [28,29]. Nevertheless, the extent and consistency of anterior and caudal spread remain debated, and cadaveric and imaging investigations have demonstrated considerable variability, which may explain heterogeneous clinical performance in procedures involving wider or lower thoracic distributions [30,31].

Evidence supporting ESPB in breast surgery has expanded, with randomized and observational studies reporting reductions in postoperative pain scores and opioid consumption, along with a low incidence of complications [12,13]. However, most available data concern mastectomy and implant-based reconstruction. LD flap reconstruction poses a distinct analgesic challenge due to the combined involvement of anterior and posterior thoracic regions. In this context, donor-site pain may significantly influence respiratory function, sleep quality, and shoulder mobility, underscoring the need for analgesic strategies that address both components [23].

This clinical complexity has prompted interest in combined interfascial approaches. The serratus anterior plane block, first described by Blanco et al., provides lateral thoracic wall analgesia and has been successfully applied in LD flap reconstruction, with reports of reduced perioperative opioid requirements and improved patient comfort [17,20]. In addition, parasternal and anterior chest wall blocks, such as the PIP block, may target parasternal and medial anterior thoracic pain more directly and may complement ESPB in selected cases where anterior discomfort is anticipated [32].

To date, literature specifically addressing fascial plane blocks in LD flap reconstruction remains limited and heterogeneous, consisting mainly of case reports and small case series. Santonastaso et al. reported effective postoperative analgesia using ultrasound-guided ESPB in LD flap reconstruction without block-related complications [33]. Yamane et al. described the use of continuous ESPB for delayed LD flap reconstruction in a patient receiving antiplatelet therapy, highlighting both feasibility and safety when neuraxial techniques may be less desirable [34]. Similarly, Siow et al. reported favorable analgesic outcomes and opioid-sparing effects with ESPB in breast flap reconstruction, supporting its potential role in reconstructive practice [19]. More recent observational studies have described combined approaches that incorporate ESPB with other interfascial techniques to optimize analgesic coverage, although standardized protocols and comparative trials remain lacking [20].

Recent comparative data in LD flap reconstruction suggest that both ESPB and PVB may reduce length of stay and opioid consumption compared with local infiltration alone, further supporting the rationale for regional anesthesia in this setting [35].

A recent practical review identified 68 publications (including 31 randomized trials) supporting ESPB as a safe and reliable option in breast surgery, with consistent reductions in pain and opioid requirements compared with non-blocked controls [36].

Against this background, the present case series adds clinically relevant real-world data supporting ESPB as a core component of regional anesthesia in LD flap breast reconstruction. In our cohort, ESPB within a multimodal analgesic pathway was associated with low early postoperative pain scores at rest and during mobilization, minimal need for rescue opioids, and no block-related complications. In selected patients, an adjunct anterior chest wall block (PIP) was added based on anatomical considerations and anticipated pain distribution. Importantly, this selective use reflects an individualized, anatomy-driven approach rather than a routine combination strategy, and no conclusions regarding additive analgesic efficacy can be drawn from this small subgroup.

The observed analgesic outcomes likely reflect the combined effect of regional blocks and multimodal systemic analgesia, including intraoperative opioids, paracetamol, NSAIDs, and dexamethasone.

Remifentanil was titrated intraoperatively according to hemodynamic response and BIS monitoring. Given its short context-sensitive half-life, it is unlikely to have influenced pain scores beyond the immediate postoperative period. Nevertheless, intraoperative opioid administration represents a potential confounding factor inherent to multimodal anesthesia.

A particularly relevant contribution of this case series is the inclusion of mid-term follow-up at 6 and 12 months. Chronic pain and shoulder dysfunction are well-recognized sequelae following LD flap reconstruction and may significantly impair long-term quality of life [24]. In our cohort, no patient reported persistent pain requiring ongoing analgesic therapy, shoulder mobility was preserved, and overall satisfaction was high. Although causality cannot be established in an uncontrolled case series, these findings suggest that ESPB-centered multimodal analgesia may positively influence longer-term recovery trajectories and favorable preliminary functional and pain-related outcomes, consistent with ERAS principles [25,26].

The results of this study should be interpreted in light of several limitations. The small sample size and single-center design limit generalizability, and the absence of a control group precludes direct comparison with alternative regional or systemic analgesic techniques. In addition, follow-up outcomes were assessed through structured telephone-based patient-reported interviews, which may be subject to recall bias and did not include objective functional measurements. Finally, longer-term outcomes beyond 12 months were not evaluated.

From a practical perspective, ESPB is an attractive alternative to TEA and PVB owing to its relative technical simplicity and favorable safety profile, particularly in patients for whom neuraxial techniques are less desirable [8,9,26]. The selective addition of adjunct blocks tailored to surgical anatomy and pain distribution may further optimize analgesic coverage in complex LD flap reconstructions involving multifocal pain sources [17,27,32,37]. Overall, although the current evidence base remains limited, our findings support ESPB-centered multimodal analgesic strategies as feasible, reproducible, and clinically promising options for LD flap breast reconstruction, warranting confirmation in larger, prospective, comparative studies.

Limitations

This observational case series has several limitations that should be acknowledged. First, the small sample size and single-center design limit the generalizability of the findings. Second, the absence of a control group precludes direct comparisons with other regional or systemic analgesic techniques. Intraoperative opioid administration was not standardized using morphine milligram equivalents, and therefore, analgesic outcomes cannot be attributed solely to the regional block. In addition, validated neuropathic pain scales were not routinely applied in the early postoperative phase, as the primary focus was on acute nociceptive pain assessment. Third, postoperative pain assessments were derived from routine clinical documentation, and mid-term outcomes were collected through structured telephone-based patient-reported interviews, which may be subject to recall and social desirability bias, and did not include objective functional or validated assessments of neuropathic pain. Fourth, although follow-up at 6 and 12 months provides insight into pain persistence and functional recovery, longer-term outcomes were not evaluated. Finally, the selective use of adjunct blocks was based on anatomical considerations rather than a predefined protocol, limiting subgroup analyses.

Heterogeneity in local anesthetic agents, adjuvants, and block combinations further limits the ability to identify the contribution of individual components. In addition, opioid consumption was not standardized using morphine milligram equivalents, limiting comparison with other studies.

Future multicenter studies using standardized assessment tools and comparative designs are needed to confirm these findings and better define optimal regional anesthesia strategies for LD flap breast reconstruction.

## Conclusions

In this retrospective observational case series, the ESPB, incorporated within a multimodal analgesic strategy, was associated with favorable early postoperative pain profiles, minimal rescue opioid requirements, preserved shoulder mobility, and high patient satisfaction following LD flap breast reconstruction. Given the small sample size, absence of a control group, and potential intraoperative confounding factors, these findings should be interpreted as exploratory and hypothesis-generating rather than confirmatory. The selective use of adjunct anterior chest wall blocks reflects an individualized, anatomy-driven approach that may contribute to comprehensive analgesic coverage in this surgical setting. Larger prospective, controlled, and multicenter studies are warranted to clarify the independent contribution of ESPB within standardized perioperative analgesic protocols.
